# Identification of novel genetic variations affecting osteoarthritis patients

**DOI:** 10.1186/s12881-016-0330-2

**Published:** 2016-10-10

**Authors:** Mamdooh Abdullah Gari, Mohammed AlKaff, Haneen S. Alsehli, Ashraf Dallol, Abdullah Gari, Muhammad Abu-Elmagd, Roaa Kadam, Mohammed F. Abuzinadah, Mazin Gari, Adel M. Abuzenadah, Kalamegam Gauthaman, Heba Alkhatabi, Mohammed M. Abbas

**Affiliations:** 1 Sheikh Salem Bin Mahfouz Scientific Chair for Treatment of Osteoarthritis by Stem Cells, King Abdulaziz University, Jeddah, Kingdom of Saudi Arabia; 2 Center of Innovation in Personalized Medicine, King Abdulaziz University, Jeddah, Kingdom of Saudi Arabia; 3 Department of Hematology, Faculty of Medicine, King Abdulaziz University Hospital, King Abdulaziz University, Jeddah, Kingdom of Saudi Arabia; 4 Department of Orthopedic Surgery, Faculty of Medicine, King Abdulaziz University, Jeddah, Kingdom of Saudi Arabia; 5 Centre of Excellence in Genomic Medicine Research, King Abdulaziz University, Jeddah, Kingdom of Saudi Arabia; 6 Department of Medical Laboratory Technology, Faculty of Applied Medical Sciences, King Abdulaziz University, P.O. Box 80216, Jeddah, 21589 Kingdom of Saudi Arabia

**Keywords:** Osteoarthritis, Whole-exome sequencing, Single nucleotide variants

## Abstract

**Background:**

Osteoarthritis (OA) is a progressive joint disease characterized by gradual degradation of extracellular matrix (ECM) components in the cartilage and bone. The ECM of cartilage is a highly specified structure that is mainly composed of type II collagen and provides tensile strength to the tissue via aggrecan and proteoglycans. However, changes in the ECM composition and structure can lead to loss of collagen type II and network integrity. Several risk factors have been correlated with OA including age, genetic predisposition, hereditary factors, obesity, mechanical injuries, and joint trauma. Certain genetic association studies have identified several genes associated with OA using genome-wide association studies (GWASs).

**Results:**

We identified several novel genetic variants affecting genes that function in several candidate causative pathways including immune responses, inflammatory and cartilage degradation such as SELP, SPN, and COL6A6.

**Conclusions:**

The approach of whole-exome sequencing can be a promising method to identify genetic mutations that can influence the OA disease.

## Background

Osteoarthritis (OA) of the knee, which is characterized by gradual degradation of articular cartilage and subchondral bone, is the most common joint disease occurring worldwide [1, 2]. Approximately 27 million individuals in the USA alone are estimated to be diagnosed with OA which affects about 10% of adults over age 55 years old [2, 3]. Various risk factors have been associated with OA including genetic predisposition, obesity, gender, mechanical injuries, physical workload, and joint trauma. However, age is the most common factor and affects especially the weight bearing joints, which is often under mechanical stress [4]. The progression of OA is a consequence of mechanical, biological, biochemical, and molecular factor interactions. What correlation? This correlation can disturb the normal series of synthesis and degradation of articular cartilage chondrocytes and extracellular matrix (ECM) components, and subchondral bone [5, 6]. In normal cartilage ECM there is a balance between synthesis and degradation that sustain dynamic equilibrium which can be disrupted if microenvironment changes in the ECM composition and structure result in type II collagen loss [7].

Inflammatory stimuli are associated with cartilage ECM changes in OA and play an essential role in the pathogenesis of synovium inflammation and cartilage degeneration through inducing the catabolic activities of chondrocytes [8, 9]. These Inflammatory factors and cytokines include interleukins family (IL) (IL-1, IL-4, IL-6, IL-17, IL-18), and tumor necrosis factor- ∝ (TNF- ∝) [10–14]. For instance, the inflammatory cytokine IL-1β is highly expressed in OA stimulating the expression of matrix degradation enzymes including MMP-1, MMP-3, and MMP-13 in chondrocytes and inhibiting ECM synthesis by decreasing the expression SOX9 [15, 16]. This response ultimately decreases the expression of collagen type II and aggrecan in articular cartilage that lead to reducing the production of chondrogenic ECM [7].

The genetic predisposition to OA is not fully understood. A number of family studies have investigated the genetic nature of OA. Familial mutations were identified in the type II collagen gene (COL2A1) [17]. Mutations affecting collagen IX gene, and COL9A1 have been linked to OA of the hip [18]. Recently, various genetic association studies have identified several genes associated with OA using genome-wide association studies (GWASs). These genes include GDF5, DUS4L, Ch7q22, MCF2L, and GNL3 [19–22]. However, the genetic association between single nucleotide polymorphisms and OA disease remain controversial.

In this study, a pilot whole-exome sequencing analysis was performed on osteoarthritis cases in order to identify candidate gene mutations associated with OA in Saudi Arabia.

## Methods

### Patients

Peripheral blood was obtained from five end-stage osteoarthritis patients with age range of 46–70 years old. The collection of these samples were approved by the King Abdulaziz University Hospital’s ethical committee (No.11–557). Informed consent was collected from all donors prior to participation in this study.

### Whole- exome sequencing

Genomic DNA was extracted from peripheral blood samples of five OA cases using QIAamp DNA blood mini kit according to the manufacture procedure (Qiagen Inc, USA). Briefly, three micrograms of genomic DNA were fragmented using the Covaris S2 system. Exome capture was performed using the Agilent’s SureSelect Whole-Exome Enrichment kit (v4). Fragment libraries were prepared from the captured exomes for sequencing on the SOLiD 5500 platform (Applied Biosystems). Fragments were sequenced in single reads of 50 bp. Reads were aligned to the UCSC hg19 reference sequence of the human genome using the LifeScope analysis pipeline which was also utilized for variants identification and annotation.

### PCR amplification and sanger sequencing

DNA was amplified using custom oligonucleotides primers listed in Table 1. Polymerase chain reaction reactions (PCR) was performed to amplify the selected primers. The PCR reactions were conducted using Go Taq Green Master Mix (Promega, USA). The reactions conditions were performed at one denaturation cycle at 95^o^ C for 15 min followed by 35 cycles of denaturation at 95^o^ C for 30 s, and annealing at 52^o^ C for 30 s and extension at 72^o^ C for 30 s. The final step was done at 72^o^ C for 10 min. To perform Sanger sequencing, purification of PCR products using ethanol precipitation were required prior to the cycle sequencing reaction. Purified PCR products were labeled with BigDye Terminator kit v3.1 (ABI Biosystem, USA) for cycle sequencing according to the manufacture procedure. After the second purification for the PCR products, the sequencing analysis was performed on the automated sequencer from Applied Biosystems 3500 DNA Analyzer.

**Table 1:** Primers used for validation of the single nucleotide variations identified by whole-exome sequencing

Gene	Forward primer (5′→3′)	Reverse primer (5′→3′)
SELP	CCTGTGTAACACAATGCG	CTACAGCTCCTACTACTG
FIGNL1	ATGGTAGCAATGTCAGCAG	GAAGATCGTATCCTAGTGG
SPN	CTCGTGCTGAGCAGAGGC	CATCCTCACTGGCCACCAG
USP36	CTCGTGCTGAGCAGAGGC	CTCACTGGCCACCAGTGG
TNRC6B	TCTGGCAGAGAACAGGCTC	CACCTGTTATTGTTGTCC
HSPG2	GACTCTGCTATGCCATGTC	CACAGGTGGTCAGCGACAC
SUSD5	AGGTGTCTGCTTGACAGTG	CTAGTTACTCCTGATCAGC
ITGA8	GGAAGGCTAATAACGATCAG	GGTTAGTAACGTGTGTTCTC
PXN	CATCCAAGAGACTCTCCAC	CCATCTGCTGACCTCTAG
NLRP6	AGCAACTGGAGCTTCGTG	GTGGCGCTCGATGTCGCGC
COL2A1	CTTAGTCCAGAGACTGCG	GGCCTGACAGGTCAGCTG
COL6A6	CATCGGTGCTGCACTCAG	CTGTGGTACAGATGTTGCG
GAS6	GGCACGCAGCAGATGCAG	CCCAGGCTGGTAGCTGAG

## Results

**Fig. 1:**
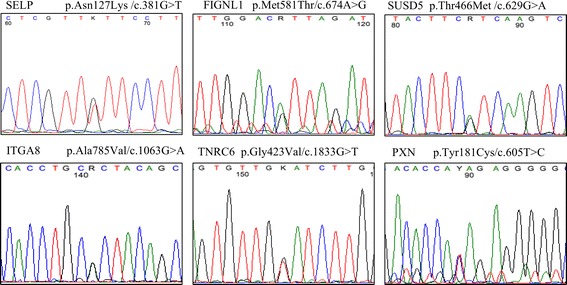
Selected chromatograms showing the Sanger confirmation of identified variants in our samples

Whole-exome sequencing of 5 OA patients revealed several novel mutations that are predicted to be damaging to protein function (SIFT). Identified variants were prioritized according to their ontology grouping in ECM and/or inflammation (Table 2). A novel c.381G > T, p.Asn127Lys mutation in the gene selectin P (SELP) has not been reported in dbSNP, 1000 Genomes project or the ExAC project. We have validated this mutation by Sanger sequencing and confirmed its existence in the heterozygous state in one patient (Fig. 1). Another novel mutation was identified in the collagen type VI alpha 6 (COL6A6) gene in another OA patient. This heterozygous c.2263T > C, p.Ile1137Thr change was also validated by Sanger sequencing. Other single nucleotide variants were identified that had no minor allele frequency reported by dbSNP. However, they were also found by the ExAC project albeit at a very low frequency (Fig. 1). These genes were identified that impact cartilages extracellular matrix (ECM) organization and cartilage development including heparan sulfate proteoglycan2 (HSPG2), sushi domain containing 5 (SUSD5), integrin subunit alpha 8 (ITGA8), paxillin (PXN), collagen type II alpha 1 (COL2A1), and fidgetin-like 1 (FIGNL1). Moreover, our results revealed some mutations associated in the inflammatory response and immune response such as pyrin domain containing 6 (NLRP6) and growth arrest specific 6 (GAS6); ubiquitin specific peptidase 36 (UPS36), sialophorin (SPN), and trinucleotide repeat containing 6B (TNRC6B) respectively (Table 2).

**Table 2:** Single nucleotide variants identified in our whole-exome screen of OA patients and validated by Sanger sequencing

Gene	Chromosome position	AA change	Nucleotide change	Protein ID	dbSNP	ExAC frequency
HSPG2	1,22154535,G,A	p.Arg4174Cys	c.4391C > T	ENSP00000363827	rs199899258	0.00005066
SUSD5	3,33194727,G,A	p.Thr466Met	c.629C > T	ENSP00000308727	rs377664152	0.00009122
ITGA8	10,15628601,G,A	p.Ala785Val	c.1063C > T	ENSP00000367316	rs371802080	0.0000248
PXN	12,120660505,T,C	p.Tyr181Cys	c.605A > G	ENSP00000267257	rs371118243	0.0001199
COL2A1	12,48371204,G,A	p.Arg1058Cys	c.1487C > T	ENSP00000369889	rs148350640	0.00002493
COL6A6	3,130293232,T,C	p.Ile1137Thr	c.2263T > C	ENSP00000351310	rs200274210	0.006855
FIGNL1	7,50513244,A,G	p.Met581Thr	c.674T > C	ENSP00000349356	rs200453649	0.000173
USP36	17,76803235,T,C	p.Arg631Gly	c.1123A > G	ENSP00000441214	rs112843316	0.000082
SPN	16,29676061,C,T	p.Arg338Cys	c.400C > T	ENSP00000353238	rs200681097	0.000485
TNRC6B	22,40661502,G,T	p.Gly423Val	c.1833G > T	ENSP00000401946	rs201057205	0.0003241
SELP	1,169586366,G,T	p.Asn127Lys	c.381G > T	ENSP00000263686	NA	NA
NLRP6	11,281256,G,T	p.Ala508Ser	c.892G > T	ENSP00000309767	rs373174851	0.000101
GAS6	13,114535300,G,A	p.Pro415Leu	c.721C > T	ENSP00000349962	NA	0.0000353

## Discussion

To identify the predisposition genetic factors and the association of these candidate genes with the disease, we conducted a pilot whole-exome sequencing on 5 OA patients from Saudi Arabia. The result of the exome sequence analysis identified several nucleotide variants affecting candidate genes that could likely contribute in OA susceptibility and involved in crucial pathways including cartilage degradation and development, inflammatory, and immune response [5].

The finding of mutations in these genes functionally interrelated to cartilage ECM organization and cartilage signaling pathway may have functional consequences. One of the identified mutations in this study is the p.Arg4174Cys mutation found in the HSPG2 gene. This gene plays multiple roles in the extracellular matrix organization involved in cartilage development, endochondral bone morphogenesis, and chondroitin sulfate metabolic process. Additionally, it is responsible for glycosaminoglycan (GAG) biosynthetic and catabolic process that maintains the expression and aggregation of mature chondrogenic markers [23]. SUSD5 harbors the p.Thr466Met mutation in one OA patient from our cohort. This gene influences the hyaluronic acid binding, a core component in articular cartilage [24]. Another mutation was identified is the p.Ala785Val mutation in ITGA8, and the p.Tyr181Cys mutation in PXN gene. Both ITGA8 and PXN genes are important in cellular matrix adhesion, ECM organization, and transforming growth factor beta receptor regulation [25, 26]. Most importantly, SNPs variants in cartilage related genes that impacts cartilage development, cartilage condensation, ECM and collagen fibril organization, and collagen catabolic process has been associated in our OA patients. That includes the p.Arg1058Cys mutation in COL2A1, and the p.Ile1137Thr mutation in COL6A6. In addition, the results of this study showed the p.Met581Thr mutation in FIGNL1 gene; a gene that influences the regulatory role in osteoblast proliferation and differentiation [27].

The result of this exome analysis revealed other mutations related to the inflammatory response such as NLRP6 and GAS6; and immune response such as USP36, SPN, and TNRC6B. The identified mutation in UPS36 gene is p.Arg631Gly. This gene is a known member of the ubiquitin specific protease family that regulates the immune signaling pathway [28]. The p.Arg338Cys mutation in SPN was also identified in our patients, and the influence of this gene can affect cellular defense responses and the regulation of the tumor necrosis factor (TNF) pathway [29]. The mutation found in TNRC6B gene is p.Gly423Val; this gene not only act in the innate immune response, but also regulates fibroblast growth factor receptor signaling pathway [30]. Furthermore, the results showed some other mutations involved in the inflammatory response. Interestingly, we identified a novel SNV variation in the SELP, p.Asn127Lys, which has not been reported previously. The function of SELP is to stimulate leukocytes adhesion in the site of injury, which could explain the presence of inflammatory markers IL family, and TNF in OA patients [31]. The p.Ala508Ser mutation in NLRP6 gene, and the p.Pro415Leu mutation in the GAS6 gene were identified in our screen; both genes regulates the inflammatory response [32, 33]. However, GAS6 specifically regulates tumor necrosis factor-mediated signaling pathway and macrophage cytokine production [33].

## Conclusions

OA is a progressive joint disease characterized by gradual degradation of (ECM) components in the cartilage and bone. Several risk factors have been associated with OA such as genetic predisposition and molecular factors. In this study, a pilot whole-exome sequencing was performed and we identified several genes candidate genes associated with OA in Saudi Arabia that function in various causative pathways including immune response, inflammatory and cartilage degradation. Despite the small sample size of this group, a hypothesis could be formulated that states the importance of personalized genetic screening of OA patients in order to understand the individual’s genetic makeup potentially responsible for susceptibility towards OA.

## Abbreviations

OA: Osteoarthritis
ECM: Extracellular matrix
SNPs: Single nucleotide polymorphisms
